# M. Alibert on Prurigo Formicans

**Published:** 1823-03-01

**Authors:** 


*Quelques Considerations sur le Prurigo Formicans.* Par M. Alibert, premier Medecin Ordinaire du Roi, Medecin en Chef de l'Hopital St. Louis, &c.*

Of all the organs of the human body, the skin presents not only the most extended surface, but the greatest variety of sensations, and consequently of pains. It is on this account, that each cutaneous malady produces its own mode and degree of suffering—but prurigo outstrips them all, in the intolerable misery which it inflicts on its unhappy victim.

This dreadful disease attacks all ages, but especially the two extremes of life—it spares no constitution—respects no class of society. Crowned heads have suffered from this terrible scourge. Men of letters, artists, and those of the legal profession, have, in M. Alibert's experience, been those most subject to prurigo, especially in the decline of life. Our author has seen it congenital, and continue during the whole lives of the unfortunate patients. He has seen three brothers in one family condemned to this malady for life! An inexperienced surgeon who had been first called in, mistook the disease for itch, and lavished his "pomades anti-psoriques," which only exasperated the evil. It is of great consequence, M. Alibert observes, not to commit this mistake. The various distinctions which authors have made of prurigo, M. Alibert is not inclined to adopt, or even admit. It has received clas-

* Annuaire Medico-Chirurgical, Vol. I.

sifications according to its degrees of force—according to the periods of life when it occurs—and, even, according to the parts where it is situated. Hence the terms mitis, ferox, infantilis, senilis, prurigo podicis, &c. which have been assigned to the varieties of the disease. M. Alibert divides prurigo into two varieties only—P. *formicans* and P. *pedicularis*. It is to the *former* variety he here directs his attention, reserving the latter for a memoir in the second volume of the work before us.

*Description of P. Formicans.* No language can convey a complete idea of the sufferings which M. Alibert has witnessed from this disease, within the walls of St. Louis. Every minute of the day and night the patient is a prey to this indomitable *pruritus*—the characteristic feature of the complaint. A devouring fire seems to consume him; and to allay the irritation he tears the skin with his nails—vain effort! the pruriginous sensation is redoubled—and some are thrown into a state of delirium by the inexpressible itching. One man put a pistol to his head, unable, any longer, to support the weight of existence.*

In most other diseases, time reconciles us, in some measure, to pain, and blunts its acuteness. Not so in prurigo. Time produces no alleviation. Nothing, but some important corporeal occupation, can at all render the patient less sensible to the irritation. Solitude and reflection increase it ten-fold. There is no other sensation that so well conveys an idea of the irritation produced by prurigo, than that of a million of ants swarming over the skin. In this complaint, there is usually an exacerbation of the burning and itching at bed-time, and at three o'clock in the morning. At the latter period, however sound the sleep, the patient is suddenly awoke, and his nails go unconsciously to work in tearing the irritated surface. The expressions which patients sometimes make use of to des-

* It has often struck us as a remarkable fact, how rarely we see suicide committed by those who labour under the most painful and tedious diseases, as cancer, calculus, and organic diseases of internal organs; while we, every day, see the said dreadful crime committed, by those who have no real suffering to endure, or only some slight mental inquietude to provoke the act. We think, that this, in itself, is a proof that some intellectual aberration is always at the bottom of suicide. No animal commits this crime—nor man neither, while he obeys the impulses of Nature and Reason. In those countries where a horrible superstition induces men, and women too, to devote themselves to voluntary death, Nature and Reason are *overcome* by Fanaticism for the time; or, as in the case of the Hindoo wife, the fear of ignominy triumphs over the fear of death, which all Nature, more or less, abhors.

cribe their sufferings, are very remarkable. One man compared his situation to that of the martyrdom of St. Lawrence, while grilling on the gridiron! "Dans un tel état d'irritation, les organes meme qui sont muets dans un age aussi avancé, entraient dans une érection forcée, d'ou il resultait des pollutions involuntaires."

In general, the prurigo declares itself at first, by an ardent itching between the shoulders, over the sternum, anus, abdomen, and thighs. The patient begins to scratch; but the more he scratches the more intense becomes the itching. On examining the parts at this time, there will be found a number of exceedingly small papulæ, slightly accuminated, but containing no fluid in their interior. These, when scratched, become covered with a little scaly crust the size of a pin's head, and of a brown or blackish colour. This crust, which is detached in a short time, appears to be formed by a slight sanguineous or serous exudation, elicited by the friction.

The intensity of the pruritus varies considerably according to circumstances. It is more pungent when the person is warm—in the evenings—night—after eating—after working. Even the friction of the clothes is generally sufficient to re-excite the itching, after a temporary cessation. This disease has, sometimes, intermissions of three or four hours—especially while the person is eating, or engaged in any laborious occupation. Sometimes the intermission lasts but five or six minutes. Our author knows a man, 55 years of age, and otherwise robust and healthy, who is subject to prurigo in the soles of the feet. This affection seizes him so suddenly, and so completely masters him, that, whether he be walking the streets, or in the midst of company, he is forced instantly to strip off shoes and stockings, and rub his feet against any thing that is near him. This proceeding he is unable to resist, were he in the presence of Napoleon or the grand Monarque! He knows another patient similarly affected, who finds no ease excepting by walking about till he is quite fatigued. When these paroxysms take place, he roams about the fields and high-roads like a crazy person. He has got the name of the "*wandering jew*," in consequence. The most distressing prurigo is that which attacks the genital organs in both sexes. It is accompanied by a host of secondary symptoms, which are variously modified, according to the idiosyncrasy of the individual. When it attacks the clitoris, it is a dreadful disease indeed. Our author knew an unhappy female who could obtain no respite from this affliction, but by keeping cloths, constantly wetted with the coldest water, to the parts.

When prurigo attacks old people, it is an inexorable malady. In them, he has known it produce tinnitus aurium, weakness of sight, cramps, lassitudes, spasms in the stomach and other parts, tension of the epigastrium, derangement of the digestive organs, emaciation, and a state of cruel despair. Some of these experience a temporary mitigation of their sufferings while gorging themselves with food, or swallowing alcoholic liquors, immediately after which, their remorseless enemy again assails them.

In prurigo formicans, the muscles are sometimes so irritated, that they swell, stiffen, and become so prominent under the skin, as to be distinctly traced on the upper and lower extremities. But it is more particularly on the lymphatic system that the ravages of the disease are directed. Most of these unfortunate patients sink, ultimately, under serous infiltrations, which take place in all parts of the system. A man of the name of John Mazuc, was lately in the St. Louis, for this complaint. He was in the 65th year of his age, and had always led a wretched kind of life. During the last eighteen months, he had been afflicted with prurigo formicans, in a violent degree, especially about the shoulders, armpits, and front of the chest. The pruritus ceased suddenly, in consequence of an unexpected chagrin of mind. All at once, his arms, thighs, legs, and face became tumefied—his breathing oppressed—and his bowels affected with diarrhœa. To these succeeded alarming faintness; but the symptoms were relieved by the prompt application of blisters. Three days afterwards, the prurigo re-appeared, and the tumefactions subsided. He found himself so well in a few days, that he left the hospital; but died shortly afterwards of hydrothorax.

The effects of prurigo on the intellectual system are not less remarkable. There has been, for a long time past, at the St. Louis, a man, in whom prurigo alternates with mania. When he first entered the hospital, he was in his right senses; but, then his whole body was covered with the pruriginous eruption. One morning, it was observed that his skin was become quite natural, and completely free from a chronic eruption of long standing—but he was so delirious, that it was necessary to confine him by means of a strait waiscoat. He laughed immoderately—asserted that he was a literary character of great celebrity, and that his name was VOLTAIRE. When the skin becomes re-affected the maniacal hallucination disappears.

The *terminations* of prurigo are not always the same. When the disease is not very intense in degree, and when it affects the tender skin of females and children, it often vanishes, without leaving any trace of its previous existence. But if the disease has continued long on a hard and tough skin, as on that of old people, the epidermis becomes exfoliated, like the skin of a serpent, or acquires a coriaceous density—a sure sign of an incurable disease.

M. Alibert gives the particulars of three dissections of this disease; but the only phenomena peculiar to the prurigo, were serous infiltrations in various parts of the body. The other morbid lesions could not, we think, have any connexion with the disease under consideration.

Our author justly observes, that it is the melancholy privilege of man, to transmit to his descendants his corporeal infirmities. Almost all the cases of prurigo formicans, in M. Alibert's experience, could be traced to an hereditary taint. He observed, that those of fair, transparent, and diaphanous skins, are more liable to this disease, than those whose skins are brown, whose muscular fibres are vigorous. Our author has been able to ascertain, as far as proofs in medicine can be obtained, that the production, or, at least, the development, of prurigo, is sometimes owing to suppression of natural discharges—especially the menstrual. He met with one case of *intermittent* prurigo in an infant, the accessions of the disease taking place every time the mother approached the menstrual periods.

Prurigo appears sometimes to be the crisis of another disease. A man who had been laboriously employed in haymaking, during a very hot day, was seized with pharyngeal angina, which assumed a chronic form. In three months this disease disappeared; but a pruritus was felt at the edge of the anus, to which succeeded a general itching all over the surface of the body, with the development of a crop of small tubercular eruptions under the skin. These continued a month, and then disappeared on the breaking out of bleeding hæmorrhoids.

The external causes of p. formicans are, according to Alibert, sufficiently numerous; over-exertion, fatigue, watching, &c. disturb the circulation, and frequently give rise to this terrible disease. A man, whose occupation was to conduct the flotillas of wood on the Seine, had a release from the malady, whenever he kept a few days quiet in the St. Louis; but a new accession of it, every time he resumed his employment. The same was observed in the case of a courier, who had prurigo whenever he travelled, and an intermission whenever he kept himself quiet. Living in damp and badly ventilated habitations, the abuse of spirituous liquors, the use of salted and unwholesome provisions are causes of the disease more powerful than avoidable. Almost all the male patients in the St. Louis are men of an idle disposition, who spend the greater part of their time in cabarets, and daily violate the rules of good regimen. It would appear indeed that the disease itself generates a disposition to indulge in hurtful things. Our author has known the disease called forth by vivid moral impressions. He states the case of a female who, by the death of her husband, fell from affluence to indigence. A severe attack of hæmoptysis was the first consequence of the mental affliction, and this terminated favourably by suitable treatment in six weeks. Her convalescence was slow, and interrupted by pains in her limbs, and excessive perspirations. The menses now became suppressed, and immediately prurigo formicans was developed on the trunk of the body, and the nape of the neck. The itching was dreadful; but the disease was at length removed by emollient baths.

*Treatment.* The remedial indications in this disease are founded on very uncertain bases. Art is yet in its infancy as far as regards the cure of prurigo formicans. The disease is but too often confounded with psora, and the means had recourse to are generally prejudicial. When prurigo is purely accidental—when it attacks vigorous subjects—and can be traced to evident external causes, it may be cured by emollient baths and a cooling regimen. But when it attacks old people, we are foiled. It is scarcely less obstinate when it occurs soon after birth. Our author has known many children in whom the disease persisted till the age of puberty. When prurigo is caused by the suppression of some accustomed discharge, it is much more easily cured than when owing to a more profound and inveterate origin—provided too active repellents are not employed, by which we risk the transferrence of the irritation from the perephery to the brain. Mental alienation is a common consequence of this metastasis. A young female was affected with prurigo from her infancy, which obstinately persisted, in spite of every means that could be devised. At length she was persuaded to apply the anti-psoric liquor of a celebrated medicaster of Paris. The eruption was suppressed very promptly indeed; but since that period Mademoiselle has not enjoyed one moment's repose. She is tormented with a feeling of extreme formication all over the cutaneous surface, but without any perceptible eruption. M. Alibert, at the time he wrote, was prescribing the warm sulphureous baths, with the view of restoring the papular eruption too suddenly suppressed.

M. Alibert offers a few laconic rules for the treatment of this troublesome complaint. When a patient first applies, he recommends that the primæ viæ should be well cleared—for which purpose an emetic is often a proper remedy. After purging, the patient is to be put on a course of diluent and aperient drink, as whey, barley water, &c. The regimen should be particularly attended to. Nothing but the simplest food should be allowed. All salads and spices must be interdicted. The juice of bitter and diuretic herbs is useful. The patient should go daily into an emollient bath, after the example of the ancient Romans, who used oleaginous baths. He has known children cured by daily baths of warm milk. Excepting some mild ointments, containing the precipitates of mercury, M. Alibert seems averse to all external applications.

We shall only add a few observations from Willan's costly work, which is not likely to be in the hands of the majority of our readers. From alkaline medicines he has seen some relief obtained; but he has generally prescribed sulphur internally, every morning and evening for a fortnight, in doses varying from ten grains to a drachm. In some cases the sulphuric acid may be taken with advantage, after the sulphur is left off. Dr. Willan has often prescribed the sulphur combined with the fossil alkali, (natron preparatum,) at the same time directing an infusion of sassafras, or the tops of juniper, to be drunk freely. Under this course, the symptoms are, in many cases, gradually alleviated, and the complaint disappears in a month or six weeks.

Dr. Willan seems to have little faith in any other external application than the warm bath, or the same impregnated with alkalized sulphur. Sea bathing has also, in some cases, entirely removed the complaint, as was long ago observed by the medical poet, Q. Serenus Samonicus.

> " Pruritus autem salsos levat humor aceti;
> " Sive maris rabidi sudor, cochleæque minutæ,
> " Quarum contactu perimetur acerba libido."
>
> *Precepta, cap.* 7.

---

